# 'People pull the rug from under your feet': barriers to successful public health programmes

**DOI:** 10.1186/1471-2458-8-173

**Published:** 2008-05-22

**Authors:** Deborah Ritchie, Wendy Gnich, Odette Parry, Steve Platt

**Affiliations:** 1 Nursing Studies, School of Health in Social Science, University of Edinburgh, UK; 2 Research Unit in Health, Behaviour and Change (RUHBC), School of Clinical Sciences and Community Health, University of Edinburgh, UK; 3 Social Inclusion Research Unit, Social Welfare and Community Justice, University of Wales, UK

## Abstract

**Background:**

A community public health programme, '*Breathing Space*', aimed to tackle smoking in a low income area in Scotland. This paper draws on the qualitative process evaluation of a community-based initiative '*Breathing Space*', which set out to tackle smoking in a low income area of Scotland, in order to explore user perceptions of key factors affecting implementation, and in particular to explore the implications of participant knowledge and expertise for programme stability and continuity.

**Methods:**

The overall evaluation of *Breathing Space *used a quasi-experimental design and incorporated a detailed process evaluation. The process evaluation aimed to document development and implementation of the programme using a range of qualitative methods, including observation, in-depth interviews, focus groups and documentary analysis. The paper draws upon 59 semi-structured in-depth interviews which were carried out as part of the process evaluation.

**Findings:**

Staff numbers from the multi-agency partnership dwindled across the lifecouof the programme and respondents identified lack of continuity as a key issue. While staff changes are an anticipated problem in programme implementation, here we draw on concepts of technicality and indeterminacy to explore the different aspects of public health programmes which are forfeited when individuals leave. The paper argues that, while technical components of public health programmes (such as the importance of staff complement and continuity) are widely recognised, it is the more indeterminate aspects, including the loss of key theoretical understanding underpinning the programme, which most affect programme delivery. Indeed, the paper suggests that, where inadequate planning and resources threaten the continuity of indeterminate knowledge, the success of public health programmes may be especially jeopardised.

**Conclusion:**

Community-based programmes which rely strongly on partnership processes would benefit from early consideration of the potential risks associated with both expected and unexpected stakeholder change. Building in appropriate contingency plans is necessary for sustaining the theory and culture of the programme. Evaluations of innovative community development initiatives may benefit from a formative approach.

## Background

Smoking is recognised as the largest single cause of preventable death and serious ill-health in Scotland [1]. The most recent Scottish Health Survey (conducted in 2003) found that 51% of men and 45% of women in the lowest household income quintile in Scotland smoke in comparison to 15% of men and 13% of women in the highest household income quintile [2]. Smoking, now statistically abnormal and normatively deviant in higher social class groups and communities, is overwhelmingly associated with social and material disadvantage and concentrated in areas of multiple deprivation.

A systematic review of community interventions for reducing smoking among adults synthesised findings from 37 studies [3]. The estimated net decline in smoking prevalence ranged from -1.0% to 3.0% for men and women combined. The two most rigorous studies showed only limited evidence of effect on prevalence. In addition, Platt et al [4] examined the impact of a range of cessation interventions (mostly community-based) from the perspective of their impact on socio-economic inequalities in smoking. They identified twenty-five relevant studies. Of the 16 studies targeting low socioeconomic groups, half demonstrated some degree of effectiveness. Platt et al [4] also highlighted a further nine studies which, although not targeted at low socio-economic groups, produced findings about differential impact according to socio-economic status. In five of these studies the intervention was at least as effective in low as in high socioeconomic groups, whereas in four studies the intervention was shown to be less effective in low, compared to high, socio-economic groups. This evidence suggests that the value of community-based interventions in reducing overall smoking prevalence or smoking-related inequalities remains uncertain.

Many recent community programmes tackling smoking form part of wider initiatives seeking to reduce cardio-vascular disease or cancer [5-13]. The majority of these programmes utilise some form of community organisation to form partnerships with their communities. While there has been a substantial amount of research on the effectiveness of partnership working [14-16], there have been few studies which explore obstacles to, and facilitators of, the successful implementation of specific programmes in entire communities from the perspective of participants. Thompson et al [17] noted a paucity of data on the process of engaging communities in tackling behaviour change, although community involvement was (and still is) an increasingly advocated approach to implementing health-related initiatives. Indeed, factors affecting the use and usefulness of community development approaches remain poorly understood despite an identified need to develop and disseminate this knowledge for health practitioners [18-28].

This paper draws on the qualitative process evaluation of a community-based initiative '*Breathing Space*', which set out to tackle smoking in a low income area of Scotland, in order to explore user perceptions of key factors affecting implementation, and in particular to explore the implications of participant knowledge and expertise for programme stability and continuity. This process evaluation documented and examined the early development and implementation of the programme, in order to understand whether strategies were implemented as planned and whether expected outcomes were accomplished. The findings of the outcome evaluation are described elsewhere [29]. In particular, they indicate that the *Breathing Space *programme did not achieve its intended aims. That is, there was little evidence of a major impact of the programme. Findings from the process evaluation of the project implementation not only provide important insights which help us to understand the failure of *Breathing Space*, but may usefully contribute to learning for future practice.

### The programme

**Figure 1:**
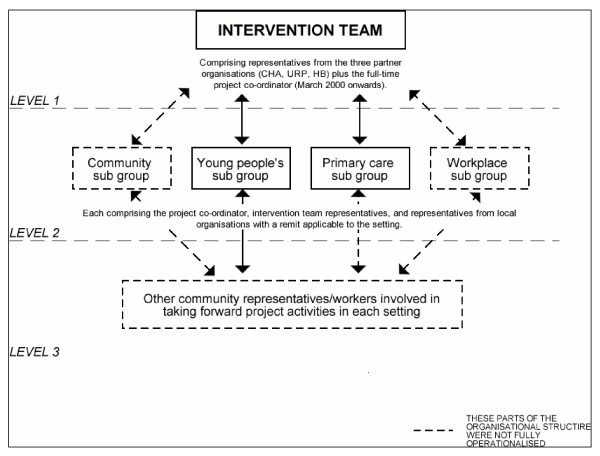
The structure of the intervention team.

*Breathing Space *was the first community-based public health programme in Scotland which specifically addressed smoking. The programme was initiated in 1998 by the local health sub-group of the local urban regeneration partnership, both of which were solely managed by local people. The group contacted the local Health Board for help in tackling the high prevalence of smoking in their local community. The management of the programme was undertaken by a partnership of the organisations run by local people, the Urban Regeneration Partnership (URP), and the community health agency (CHA) and Health Board, between 1998 and 2001 (figure 1). The local agencies, while run by local people, did employ staff to undertake the community work. The programme aimed to produce a significant shift in community norms towards non-smoking in a low-income area with a population of 22,884, and a deprivation ('DepCat') score of 5, within a large city. (DepCat is a measure of social deprivation, widely used in Scotland, which is based on information gathered in the national census every ten years and describes the socio-economic composition of residents in a particular postcode sector. DepCat scores for each postcode area in Scotland are calculated from the percentage of unemployed males, over-crowded households, households without cars and people from social classes IV and V. The scale runs from DepCat 1 (most prosperous) to DepCat 7 (least prosperous) [31]).

The conception and design of *Breathing Space *was grounded in the principles of community development, including shared participatory decision making and consensus. It was intended that intervention activity would be planned and driven by the local community, with leadership for the programme nurtured from within the community [28]. A previous paper explored the tensions and contradictions in implementing these principles in practice [19].

The programme (the content of which is captured in table 1) was delivered in three phases over a three year period: first, the mapping phase (1998–1999), when existing community activity to tackle smoking was recorded and local participants were identified and recruited; second, the planning phase (spring 1999), when findings from the mapping exercise were disseminated to the local community led groups and the interventions were developed; and, thirdly, the implementation phase (Autumn1999–Summer 2001). Programme funding was provided by the local health board, and the locally run community agencies gave contributions in time and local administrative support. Programme responsibilities were defined through the planning process, with an expectation that the local agencies would deliver the programme in collaboration with local people.

**Table 1:** Summary of intervention activity

**Setting**	**Programme activities**
**Community**	Provision of smoking cessation support and holistic healthcare as an alternative to practice based support
	Development and delivery of training programme for community workers in smoking cessation support
	Distribution of information regarding support available to those who want to quit
	Profile raising activities: community events, posters, local newspaper (adverts/competitions), post-card drop to every household
**Primary care**	Operational and strategic input into local smoking cessation planning
	Training of health professionals in brief and in-depth intervention methods inc. motivational interviewing
	Support of smoking cessation counselling services set up through Local Health Care Co-operative
	Provision of Nicotine Replacement Therapy through a community venue
**Schools/Youth**	Production of a sustainable education pack suitable for use as a teaching aid in secondary schools
	Leaflet design project and competition involving first year pupils in local secondary school.
	Clear signage about no smoking policy adopted in local secondary school
	Funded community grant projects: posters, video, web-site design, alternatives to smoking/activity groups
	Development and implementation of a protocol for the provision of smoking cessation support for those aged under 16 years
**Workplace**	Offer of health audit/support to Small and Medium Enterprises

The programme was delivered in four health promotion settings (community, schools/youth, primary care and workplaces) and comprised a range of activities, such as training health workers, young people's videos, health fairs in the shopping centre, newspaper features, and innovative smoking cessation and well-being programmes (table 1).

## Methods

The process evaluation focused upon the design, development, scope, intended purpose and implementation of the *Breathing Space *programme. To this end it used a range of qualitative data collection methods, including: observation (at programme meetings and key events); in-depth interviews (with key stakeholders including programme managers and workers); focus groups (with programme implementers and young people); and examination of official documents (minutes, reports, budget statements, policy documents and key correspondence) and monitoring of local newspapers and community publications.

The paper draws upon 59 semi-structured in-depth interviews which were conducted with programme planners and implementers, including programme managers, intervention team members and other individuals from the local community with involvement in programme activity (sub-group members) at three key phases in the life of the initiative (mapping, planning and implementation) (Table 2). Interviews were conducted in person and tape recorded, and lasted on average for one hour. They explored understandings of the programme at different levels: overall programme organisation and structure; individual projects; and personal roles and responsibilities.

**Table 2:** Interviews conducted as part of the process evaluation

**Staff category**	**Organisation/Setting**	Phase of programme*			**Total**
			
		**Mapping**	**Planning**	**Implementation**	
Project co-ordinator(s)		-	-	8	8
Intervention team**	Health Board	5	5	10	20
	URP	1	1	4	6
	HA	1	1	2	4
Subgroup members	Community	-	-	2	2
	Primary Care	-	-	5	5
	Young People	-	-	6	6
	Workplace	-	-	-	-
Management	Health Board	1	1	3	5
	URP	-	-	1	1
	HA	-	-	2	2
**Totals**		**8**	**8**	**43**	**59**

*Definiton of the programme phases

**Mapping**: *Activity undertaken as part of the needs assessment/audit. Aimed at gathering the views of key community members to assess smoking related activity and attitudes. As well as the engagement of key community participants*.

**Planning**: *Activity related to setting, revisiting and reshaping project objectives. Agreeing responsibility and leadership for specific objectives/projects and summarising/tracking current progress in order to facilitate reshaping of objectives. (Overall and subgroup level)*

**Implementation**: *Activities directly related to carrying out or facilitating specific project objectives. 'Actual doing'. Including activity related to securing appropriate funding and resources to allow specific initiatives to progress and involvement of community members or workers in order to progress the objective (undertake particular activities)*.

**Key intervention team members were interviewed three times during the implementation Phase of the programme.

Analysis of the process data was informed by a grounded theory, constant comparative approach. Interview transcripts were read and re-read by members of the process evaluation team in conjunction with data obtained from the other process evaluation research methods. Thematic categories were identified in the combined datasets and explored in order to ascertain which aspects of the intervention enhanced or hindered the successful design, development, implementation and receipt of community based programmes. Reliability of coding procedures was established through frequent meeting of team members. The robustness of both pre-identified and emergent categories was tested by reference to the individual cases, and conditions and circumstances of these formulations were compared and contrasted. A qualitative software package (NUD· IST) was used to assist in the management and combination of the combined datasets (from observation, mapping of community activity and interviews).

Extracts from data presented in the paper are prefixed by a letter which identifies the type of intervention participant: 'M' signifies manager, 'I' signifies intervention team member, and 'CP' signifies intervention member from the local community group.

### Ethics

Since the research was community-based and did not involve NHS patients or staff, it was neither necessary nor appropriate to submit the protocol for inspection to a NHS ethics committee. However, we ensured that our research practice was consistent with University of Edinburgh research ethics framework and with guidance provided by professional bodies, such as the British Psychological Society, the British Sociological Association and the Social Research Association. All respondents were provided with a written information sheet and signed consent forms.

### Findings

#### Managing change

While change is an anticipated component of community based programmes, the level of change associated with *Breathing Space *far exceeded planned resource provision. Respondents described these changes as imposed upon the programme from outside and as impacting negatively upon the programme in a number of ways.

Staff turnover and attrition constituted a major issue for respondents. Over the lifecourse of the programme, the number of individuals associated with the design, development and implementation of *Breathing Space *decreased considerably. While respondents acknowledged that "things are always going to happen within a project that's spanned over such a long period of time" (CP3), by the end of the programme they lamented that there were "few members of the original team left" (I13).

Attrition was ascribed to several factors, of which the most important was organisational change within the programme's partner organisations, both locally run and external public organisations. The local area had experienced ten years of an extensive urban regeneration funding that was coming to an uncertain end. The withdrawal of significant urban regeneration partnership personnel and resources that supported local people had serious repercussions for the *Breathing Space *project. This insecurity was further compounded by re-structuring in the Health Board. Of six original intervention team members employed by the Health Board only two retained involvement throughout the three year initiative. Moreover, of three original community intervention team members (employed by the local CHA and URP) all were either on long-term absence or resigned during the course of the programme. At a managerial level, two managers at the CHA, two at the URP and five different Health Board staff had responsibility for Breathing Space during its lifetime. All these changes were exacerbated by uncertainties over core funding in the partnership and associated staff cut-backs in all three partner organisations:

"*Each agency has transformed completely. (Health Board) went through restructuring. The Partnership (URP) is winding down towards closure and the Health Agency (CHA) have got staff shortages and are now completely, well until recently out of the loop*" (I10).

As a result of these changes, funds were not available to replace staff on long term sickness, posts were frozen and/or part-time workers replaced full-time workers. While the reasons underpinning staff turnover are important, in this paper we focus upon the implications for the integrity of the programme of the loss of these very different, and often key, contributions.

#### Programme ownership and control over decision-making

Staff turnover served to undermine the sense of control over the programme among programme staff and local people. This, in turn, eroded key stakeholders' sense of programme ownership. The significant changes in the programme managers meant that *Breathing Space *found itself without the authority to make and act upon decisions. Under these circumstances, key decisions relating to the programme often lay outside the control of key programme workers and local people, and programme staff frequently had to await decisions from others in the parent organisations:

"*I suppose bureaucracy, big organisations being what they are, they think, they obviously feel that they've got to make their own in-house decisions*" (I10).

Even when new members of management were appointed to fill the vacant posts, they did not share the same programme history. Thus, intervention team members were required to refer to line managers who did not necessarily have in-depth knowledge about the programme. Respondents reported being unsure as to which manager was responsible for what, while lacking the authority to make their own decisions.

Staff turnover/attrition was also described as impacting on the relationship between the three main partner organisations. From the outset the Health Board was perceived as the most powerful player in *Breathing Space*, with more staff and heightened visibility, particularly in the early stages of the programme. As the community regeneration partnership wound down and key community workers on the programme were absent on long term sick leave, decisions were increasingly perceived to come from the Health Board. This had implications for maintaining community involvement:

"*The partnership were the link to the people locally. They were the link to the other work that was going on. They were integrating the sustainability of the initiatives. And basically they were to take forward the community setting, which just hasn't happened*" (I5)..

#### Knowledge and expertise

As participants of the programme left and/or were replaced, knowledge and expertise which they bought with them or acquired through familiarity with the programme, were forfeited. Theoretically, new participants drafted on to *Breathing Space*, to replace those who had left, could be initiated into the programme. However, respondents perceived this as a less than ideal solution for a number of reasons.

First, those involved in the initial design and development of the programme were considered to be 'visionaries' whose intensive association with *Breathing Space *was perceived as necessary for the programme to realise its objectives. The loss of these key players was felt deeply by respondents:

"*...we've not had (visionary X) there, who we can get instant answers from, about Breathing Space, and about our interventions and things, so there's been a lack of direction*" (I7).

"*They were the key people that, if there was anything I wasn't sure about, I was able to ask. So, in these terms, in terms of sourcing information...it's a bit more difficult, I now find, to clarify things for myself. You know, other than reading through past papers*" (I4).

Second, the early stages of *Breathing Space *involved the identification of useful community local contacts and the development of local networks between these individuals. However, because these networks remained at the individual level, and were not institutionalised at programme level, the support which they provided was forfeited when the key contacts left:

"*...she would build up networks and contacts just by doing that. So we've lost that. We've definitely lost out by the fact she is moving*" (M1).

"*It's difficult for somebody else coming in who has not had the leads created*" (I2).

Third, specialist skills deemed necessary to the successful working of the programme were lost. Staff members who had "worked that way before" and who were felt to have "particular community development skills" were particularly missed:

"*I think her particular community development skills will be, she has worked in that way before, her understanding of that will be a great loss, actually*" (M1).

As original members left, information about the programme became increasingly difficult to source. A member of the intervention team who came on board during the last year of the initiative explained:

"*... it has taken me a long time, a good while to get my head around what it's all about. And I think there have been difficulties in people informing me about things because there are so few members of the original team left*" (I13).

#### Loss of continuity and loss of momentum

Respondents expressed concern that staff turnover had implications for sustaining the ethos of *Breathing Space *in its original conception:

"*...new people come in and they are picking up and they are bringing their own approach to things. But I don't think that's been maybe so significant as the fact that, because of absences in other people, even just people stepping in for a short term isn't the same for momentum as people being consistent and believing in the ethos and continuing it right the way through*" (CP1).

Furthermore, the theoretical direction of the programme became diluted and the understanding of the new programme leaders was not always consistent with the original programme objectives. The original commitment of working in and with the community was transferred to the more traditional primary care models of smoking cessation.

"*One of the big objectives has kind of almost been forgotten about... is about changing perceptions, and that we've concentrated very much on primary care and not the community. And I think that's partly through the coordinator when he took over post maybe not being given a fully accurate understanding of what the project was about and then maybe lost it again with someone else. A bit like Chinese whispers: you start off and you end up with a very diluted message*" (I6).

For the most part respondents understood these changes to be a function of the lack of programme continuity:

"*Not only (were there) not enough people but not the continuity. There's huge, huge numbers of changes from the beginning to the end. I mean, with all the people who are not involved now but were at the beginning for various reasons*" (CP1).

Loss of continuity was, in turn, associated both with staff departure and with the unequal inputs from, or the perceived value of, those contributing from different organisations. The implication here was that, if input into the programme had been more equal, then staff losses from one quarter may have been more easily accommodated.

Restructuring and associated financial cutbacks in *Breathing Space*'s partner organisations, which impinged on the amount of funding available for programme work, were also associated with a loss of momentum. Respondents talked about a "failure to follow through" on original pledges, noting that the ability of local community organisations, in particular, to execute their agreed programme of work was adversely affected.

"*It's hard to look forward because people pull the rug from under your feet all the time. One year they want a four year business plan and the next year they will be cutting twenty percent off your money*" (M5).

While community partners acknowledged their inability to fulfil the contribution they had agreed during early programme planning, they were resentful when others (particularly Health Board staff) stepped in to carry out work on their behalf.

## Discussion

The findings of our analysis clearly indicate that the Breathing Space programme may have benefited from a formative evaluation which enabled programme participants to reflect on and respond to challenges (particularly concerning community involvement) arising through implementation. While a formative approach would have been difficult to incorporate into the overall quasi-experimental design of the Breathing Space evaluation, in retrospect some feedback to programme implementers might have been possible without compromising the study.

A limitation of the process evaluation is that it is not possible to know the extent to which the findings might reflect the experiences of programme intervention in other types of communities (e.g. less socio-economically deprived). In addition, some programme effects of interventions such as Breathing Space may be unanticipated and experienced after the funding, and indeed the programme, has ended. Hence, the process evaluation was unable to capture the implications of staff changes for longer-term programme effects.

In accordance with findings from previous research we found that inadequate resource allocation and poor continuity of structures and personnel are key barriers to successful implementation of community based partnership programmes [18-20,23,31-33]. As, Backett-Milburn and Wilson [34] illustrate, high staff turnover is often found in community based programmes.

In order to understand in more depth the ways in which stability and continuity in the staff base of interventions might facilitate successful implementation, we employ the dual concepts of indeterminacy and technicality, originally used to describe particular aspects of occupational work [35-37]. Jamous and Pelouille [38] developed the concept of 'technicality' to provide a classificatory device for the professions. Technicality can be described as that part of occupational work which lends itself to documentation, and at its extreme this aspect of work can be conveyed by a list of specifications graspable through memory and physical dexterity. Juxtaposed to technicality is indeterminacy, which implies a kind of tacit, implicit knowledge which remains the personal property of the practitioner. Different types of occupational work incorporate technical and indeterminate aspects in differing degrees [38-42].

In the literature, staff attrition and turnover are recognised as major barriers to implementation, mainly in terms of technicality. That is, when staff leave the personnel resource for the programme is depleted. This may include key individuals with skills difficult to replace. Existing staff must carry out the work of those departed, as well as performing their own roles. In addition, they may be responsible for socialising replacement staff into the work of the programme.

However, it is the indeterminate aspects affected by staff attrition which are key to successful implementation. The partnership literature suggests that the early development of underlying theoretical positions, vision and shared understanding which drive interventions is essential for successful outcomes [15,16]. We suggest that loss to the project through staff attrition/replacement undermines these indeterminate aspects of implementation work. Key aspects of implementation which may be described as indeterminate are two-fold. The first constitutes knowledge about the programmes associated with key individuals, which is forfeited on their departure. Our data indicate that knowledge gained during early conceptualisation and planning of interventions, and early community engagement work, is an important resource to draw upon during subsequent programme implementation stages. When this knowledge disappears, programmes are in danger of losing direction and identity. This is particularly pronounced when departing programme members have contributed to the theoretical underpinning of the programme. Second, the loss of participants may also have repercussions for control over programme decision making, and the balance of power between partnership organisations. Where there is an existing perceived imbalance between formal agencies and groups run by local people this may become more pronounced as a result of staff changes. This has particular implications for ownership of programmes as perceived by their participating members. Moreover, it must be acknowledged that those partnership organisations, that are run by and for local people, which traditionally are perceived (and perceive themselves) as the least powerful players in an intervention partnership will have most to lose in this respect. The importance of indeterminate knowledge to programme success is clear in the case of Breathing Space, because loss of the knowledge and understanding which underpinned the original community based ethos of the programme served to undermine participant ownership and programme direction.

The findings suggest a number of concrete recommendations that could counteract the indeterminate aspects of community partnerships. First, clarity would be improved with a formal negotiation and agreement about the actual commitment of personnel, time, funding and other resources by each partner organisation, at the outset of the project. Second, contingency plans must be incorporated into the project design in order to accommodate change, and a comprehensive process of induction should be developed to ensure the original aims of the programme are transferred and understood. Third, contingency funding could be built into the resources allocated to small community-led organisations in order to help them manage sickness absence. Fourth, the inevitable change processes involved in such projects require effective management and communication between all partner organisations. Fifth, a culture of mutual responsibility and accountability should be fostered in all partner organisations. Sixth, it is important that diversity of knowledge, experiences and resources is acknowledged and valued across the partnership groups, celebrating the contribution of both lay and professional knowledges to the programme endeavour. The promotion of such inclusiveness will, it is suggested, add to the depth of understanding about the problems and generate creative and contextually relevant solutions. Seventh, the involvement of local people can be eroded when external agencies and local community agencies are unable to sustain the vision and community involvement principles due to these indeterminate aspects.

Maintaining community involvement should be considered central to success. Finally, an increased emphasis on the diffusion of 'what works' through community structures will usefully inform future intervention programmes and curb the tendency of interventions to 'reinvent the wheel'.

## Conclusion

In conclusion, while more technical aspects of staff attrition and replacement may be acknowledged in the literature, the more indeterminate aspects of programme work are crucial to understanding how particular programmes and activities may be accomplished in practice. Community programmes which rely strongly on partnership processes and the involvement of local people would benefit from early consideration of the potential risks associated with both expected and unexpected stakeholder change. Building in appropriate contingency plans is necessary for sustaining the theory and the culture of the programme.

This analysis should not lead to the conclusion that community programmes, such as *Breathing Space*, are ineffective. Rather, the programme planning was not able to focus fully on community engagement because of a failure by the programme leaders, both from the local community agencies and the external health board, to address these indeterminate aspects of the programme's implementation.

## Competing interests

The authors declare that they have no competing interests.

## Authors' contributions

DR was involved in the design and management of the project. She was involved in some of the acquisition of the data, in particular the individual in-depth interviews, focus groups and observations. She was involved in the analysis and interpretation of the process evaluation data. She has contributed substantively to the intellectual development of the paper and the writing of the final manuscript.

WG undertook data collection for the process evaluation and management of the surveys. She was engaged in both the analysis and interpretation of the process evaluation data and the survey data. She contributed substantively to the intellectual development of the paper and the writing of the first drafts and has made critical comments on the drafts of the paper.

OP was involved in the design and management of the project. She was involved in the analysis and interpretation of the process evaluation data. She has contributed substantively to the intellectual development of the paper and the writing of the first drafts, and commented on the final draft of the paper.

SP was the principal investigator on the project. He was involved in the management of the survey and the process evaluation. He contributed to the analysis of the survey data and the process evaluation, and to the intellectual development of the paper, and has made critical comments on the drafts of the paper.

## Disclaimer

The views expressed in the publication are those of the authors and not necessarily those of the Department of Health.

## Pre-publication history

The pre-publication history for this paper can be accessed here:


